# Osteosarcoma neutrophil extracellular trap network-associated gene recurrence and metastasis model

**DOI:** 10.1007/s00432-023-05577-2

**Published:** 2024-01-29

**Authors:** Hao Tang, Jiang Xie, Yu-Xuan Du, Ze-Jiu Tan, Zhuo-Tao Liang

**Affiliations:** 1 Department of Spine Surgery and Orthopaedics, Xiangya Hospital, Central South University, Changsha, Hunan 410008 People’s Republic of China; 2 National Clinical Research Center for Geriatric Disorders, Xiangya Hospital, Central South University, Changsha, Hunan 410008 People’s Republic of China

**Keywords:** NETs, Osteosarcoma, Metastasis model, Immunity, TOMM40, FH

## Abstract

Osteosarcoma (OS) is the most common malignancy in children and adolescents and has a high probability of recurrence and metastasis. A growing number of studies have shown that neutrophil extracellular traps (NETs) are strongly associated with cancer metastasis, but in osteosarcoma, genes associated with NETs that promote osteosarcoma recurrence and metastasis remain to be explored. We systematically investigated the gene expression patterns of NETs in OS samples from the GEO database. NETs molecular typing was evaluated based on NETs expression profiles, and the association between NETs molecular subtypes and immune microenvironment and metastatic features were explored. Ultimately, we constructed a signature model and column line graph associated with metastasis prediction and screened possible potential drugs for metastatic osteosarcoma. We established two different molecular subtypes of NETs, which showed significant differences in metastatic status, metastasis time, tumor immune microenvironment, and biological effects. We also constructed a NETs-related gene metastasis signature(NRGMS) to assess the expression pattern of NETs in patients to predict metastatic recurrence in osteosarcoma patients. We screened for TOMM40 and FH associated with metastatic recurrence in osteosarcoma patients. Overall, this study constructs a predictive model for osteosarcoma metastasis of NETs-related genes, which is expected to provide new insights into the metastasis of osteosarcoma.

**Supplementary Information:**

The online version contains supplementary material available at 10.1007/s00432-023-05577-2.

## Introduction

Osteosarcoma (OS) is a common bone tumor that most commonly affects the epiphysis of long bones in adolescents and young adults(Xu et al. 2022). OS is notable for its high metastasis rate, ease of recurrence and poor prognosis, and is the second leading factor of death among childhood and adolescent cancers(Hua et al. 2022; Liu et al. 2022). Currently, surgical resection, chemotherapy, radiation therapy, hormone therapy and small molecule targeted therapy are still the main treatments for osteosarcoma(Pan et al. 2020). Combination therapy has led to a dramatic increase in the survival rate of patients with osteosarcoma, with a cure rate of nearly 70% for patients with localized disease(Harrison et al. 2018). However, for patients who have developed distant metastases, the 5-year survival rate is still unsatisfactory even with high-dose adjuvant chemotherapy combined with radical resection(Huang et al. 2018). The vast majority of patients with osteosarcoma metastases die from pulmonary metastases, and their 5-year overall survival rate is less than 25%(Whelan and Davis 2018). Therefore, there is an urgent need to explore a stable prediction model that can predict metastasis for OS patients as a guide to clinical treatment options.

Neutrophils are the most abundant endogenous immune effector cells, and neutrophil extracellular traps (NETs) are the expression patterns of neutrophils in response to specific stimuli, a cell death process known as NETosis(Ireland and Oliver 2020). In general, NETs trap, neutralize and kill bacteria, fungi, viruses and parasites, and are thought to prevent the spread of bacteria and fungi, which is thought to be the mechanism by which the innate immune system protects us from infection(Brinkmann et al. 2004). Neutrophil extracellular traps (NETs) have now been shown to promote tumor growth and metastasis(Cedervall et al. 2016). The mechanisms by which NETs promote tumor growth include capturing circulating tumor cells for their proliferation and survival or altering tumor cell bioenergetics(Yazdani et al. 2019). However, the molecular mechanisms of NETs in cancer are still poorly understood, especially how NETs affect metastasis and recurrence of osteosarcoma.

High-throughput sequencing technology is widely used in the exploration of disease pathogenesis and the development of novel biomarkers(Jia et al. 2021). Many statistical models have been developed that use genomic data to accurately predict the high or low prognostic risk of cancer patients. However, models that can predict metastasis in patients with osteosarcoma are lacking. By analyzing OS gene expression profiles, we eventually constructed a model that predicts metastasis in osteosarcoma patients and performed functional annotation, immune-related signature correlation analysis, and immune infiltration analysis to fully exploit the features of our model. We finally found that TOMM40 and FH can significantly promote osteosarcoma metastasis, and the pro-metastatic ability of TOMM40 and FH was verified by in vitro experiments.

## Materials and methods

### Data and sample collection

#### Data acquisition and preliminary processing

Gene expression data, and clinical characteristics of OS samples were obtained from the GEO database (https://www.ncbi.nlm.nih.gov/geo/), and a total of 95 OS samples were obtained from GSE21257, GSE39055 and GSE39057. These three cohorts were merged to remove batch effects using the sva.R package (Leek et al. 2012), we removed samples with no clinical information, with missing values, and with metastasis or recurrence at the first diagnosis. The final number of samples that met the requirements was 81. In addition, we downloaded the raw pan-cancer mRNA matrix data, clinical data and copy number data from the University of California, Santa Cruz (UCSC) database (https://xenabrowser.net/). NETs genes were obtained from the KEGG database (https://www.genome.jp/kegg/), and after removing duplicates and deletions, we identified a total of 97 NETs genes, and the limma.R package screened 24 NETs genes that were differentially expressed in the non-metastatic and metastatic groups(Smyth 2005).

#### Tissue sample collection

We recruited 10 patients with osteosarcoma, of whom 5 were already metastatic at the time of diagnosis. Patient inclusion criteria were as follows: 1. imaging and pathological diagnosis of osteosarcoma 2. meeting the conditions for surgery 3. not treated with chemotherapy or radiation therapy. All patients signed an informed consent form and the study was reviewed by the ethics committee.

#### Cell collection and culture

The osteosarcoma cell line used in this study was SW1353, which was purchased from the American Typical Biological Resources Collection (ATCC). The cell culture medium consisted of 90% Dulbecco's modified Eagle's medium (DMEM) + 10%FBS + 1% penicillin–streptomycin. Cells were maintained at 37 °C in a constant temperature incubator with 5% CO2. The cells were detected for mycoplasma contamination using the Mycoplasma detection kit (Beyotime, China) according to the protocal.

### Unsupervised clustering of NETs genes

To identify different NETs gene expression patterns, we applied an unsupervised clustering analysis based on the expression of these 24 NETs genes. Different expression patterns of NETs genes were evaluated using the ConsensusClusterPlus.R package based on the k-means algorithm(Wilkerson and Hayes 2010), which ultimately classified OS patients into 2 different clusters of NETs. The relevant R code and documents of the ConsensusCluster algorithm can be found in Supplementary Materials (Supplementary Data Sheet 1). The dispersion of the two groups was assessed by dimensionality reduction analysis using the principal component analysis (PCA) technique(Ringnér MJNb 2008).

### Genomic variant analysis (GSVA) and immune cell infiltration analysis

To further evaluate the biological functions in different OS groupings, we used the gsva.R package to perform GSVA(Ringnér MJNb 2008). The c2.cp.kegg.v2023.1.Hs.symbols gene set and the h.all.v2023.1.Hs.symbols gene set were downloaded from the MSigDB database (http://www.gsea-msigdb.org/gsea/downloads.jsp). The top 20 biological terms with adjusted *P* < 0.05 were finally selected. Single sample gene set enrichment analysis (ssGSEA) was performed to quantitatively characterize the enrichment fraction of immune cells in the different OS NETs clusters.

### Constructing NETs-related metastasis risk scores by LASSO regression

Differentially expressed genes (DEG) screening was performed between different OS groups using the limma.R package with adjusted criteria of *P* < 0.05. We randomized OS patients 1:1 into training and test groups and used univariate Cox regression to identify DEG genes associated with the model-constructed diagnosis-to-first-metastasis time. The best features were identified using the glmnet.R package using the least absolute shrinkage and selection operator (LASSO) and multifactorial Cox regression analysis (Hastie et al. 2020). The model risk score used the following equation: Expression of gene 1 *Coef 1 + Expression of gene 2 *Coef 2 + Expression of gene 3 *Coef 3 + Expression of gene n *Coef n. We further divided OS patients into high-risk and low-risk subgroups based on the median risk score described above. Survival curves were plotted for the high-risk and low-risk groups by using the survivor.R package and survminer.R package.

### Construction of predictive column line graphs and independent prognostic analysis

Nomogram is now widely used to predict cancer prognosis(Iasonos et al. 2008). We integrated several clinical variables to construct the colinear plots, which included age, gender, and NRGMS model. We calculated columnar plot scores to predict the probability of not having metastasis or recurrence at 1, 3, and 5 years for OS patients. Calibration curves (OS curves) to assess the calibration ability of the column line graphs. We also performed univariate and multifactorial independent prognostic analyses for the variables Nomo prediction model, NRGMS model, age and gender. The index of concordance was used to assess the predictive power of the models.

### Evaluation of immune infiltration characteristics and treatment of NRGSM

The exploration of the tumor immune microenvironment plays a crucial role in tumor immunotherapy. To assess the immune cell composition in OS samples, we performed a CIBERSORT analysis of expression data to calculate the abundance of various immune cells in the tumor microenvironment (Newman et al. 2015). The relevant R code and documents of the CIBERSORT algorithm can be found in Supplementary Materials (Supplementary Data Sheet 2). Correlation analysis between NRGMS model genes and immune cell abundance was examined by the Spearman rank correlation test. Wilcoxon rank sum test to detect differences in various immune cells and immune checkpoints between high and low-risk groups for OS. estimate.R package of ESTIMATE algorithm further calculates the estimated volume, immune and stromal scores for high and low-risk groups (Yoshihara et al. 2013). Finally, we predicted chemotherapy response in OS patients using the R package pRRophetic.R package (Geeleher et al. 2014).

### Pan-cancer analysis of TOMM40 and FH

We further investigated the role of TOMM40 and FH in tumors, assessed the differential expression of TOMM40 and FH in pan-cancer, and we performed correlation analysis of TOMM40 and FH with tumor mutational load (TMB) and microsatellite instability (MSI) in patients. In addition, we performed the co-expression analysis of TOMM40 and FH with immune cells.

### RNA extraction and quantitative polymerase chain reaction (qPCR)

We further evaluated TOMM40 and FH expression levels in metastatic and non-metastatic osteosarcoma tissues. The osteosarcoma tissues were derived from osteosarcoma patients after surgery. All subjects signed an informed consent form, and the study was approved by the Ethics Committee of Xiangya Hospital, Central South University. The total RNA of tissue specimens was extracted using TRIzol, and the mRNA expression levels of TOMM40 and FH were examined after rigorous manipulation according to the previously described method. The primers used in this study are summarized in Table 1.

**Table 1:** Primers for quantitative real-time PCR (qRT-PCR)

Gene		Primer sequences (5ʹ to 3ʹ)
FH	R F	CCTTTCTGTATTGGCCTGGA GGCAAGGTGAACCCTACTCA
TOMM40	R F	AGGAATCCTCGTAGCCCACT CTCAAACTCCACACCCACCT
RN18s	R F	AGAAACGGCTACCACATCCA CCCTCCAATGGATCCTCGTT

### Western blotting

The WB experimental method was performed strictly as described by the previous authors. The primary antibodies used were as follows: GAPDH (1:5000; CST, American), FH (1:5000; CST, American), TOMM40 (1:5000; CST, American), incubated overnight at 4 °C and conjugated with secondary antibodies at room temperature for 1 h (1:10,000, Proteintech. China). The results were subsequently detected using a chemiluminescent protein detection module (Thermo Fisher Scientific, USA) and quantified using image J software.

### Scratch assay

To determine whether osteosarcoma cells undergo migratory changes, a scratch test was performed. Transfected MG63 cells were inoculated into 6-well plates. After reaching 80%—90% cell density, cells were scraped using a pipette tip perpendicular to the bottom of the plate. Data on the distance between the left and right ends of the scratches were made at 0 h, 24 h and 48 h under the microscope, respectively.

### Statistical analysis

All bioinformatics statistical analyses were performed in R software (version 4.2.1), and values of *P* < 0.05 were considered statistically significant. Student’s *t*-test (unpaired or paired) was used to determine the significance of the differences between the two groups. Statistical significance was set at *P* < 0.05. All data are expressed as mean ± standard deviation.

## Results

### NETs expression mode in OS

**Fig. 1:**
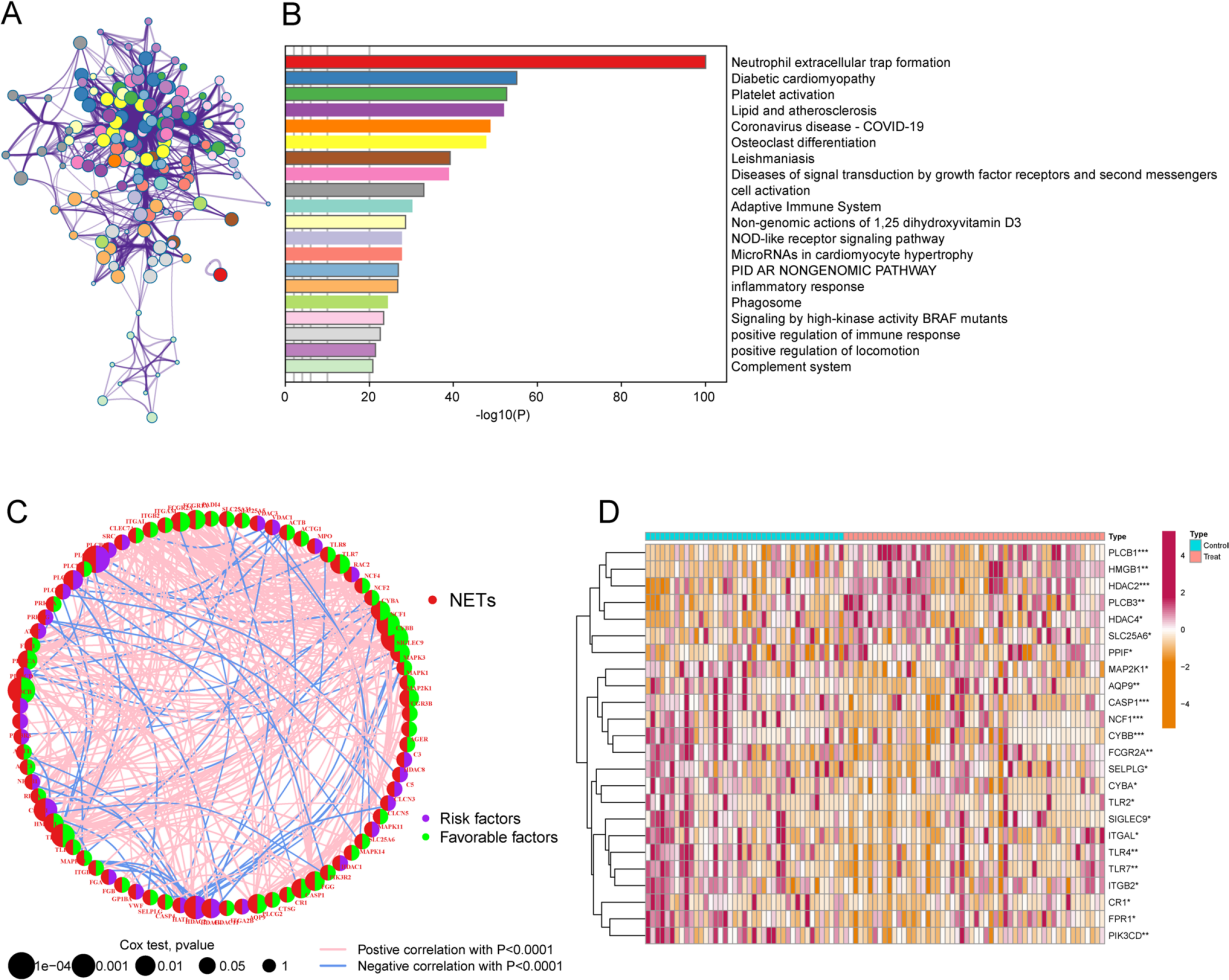
Characteristics of transfer-related NETs. **A** shows the functional pathway interactions of NETs genes, **B** shows the functional pathway enrichment map of NETs genes (showing the top 20 most significantly enriched pathways), **C** shows the interaction network of NETs genes associated with metastatic features, and **D** shows the heatmap of differences between metastatic and non-metastatic groups of osteosarcoma patients

**Fig. 2:**
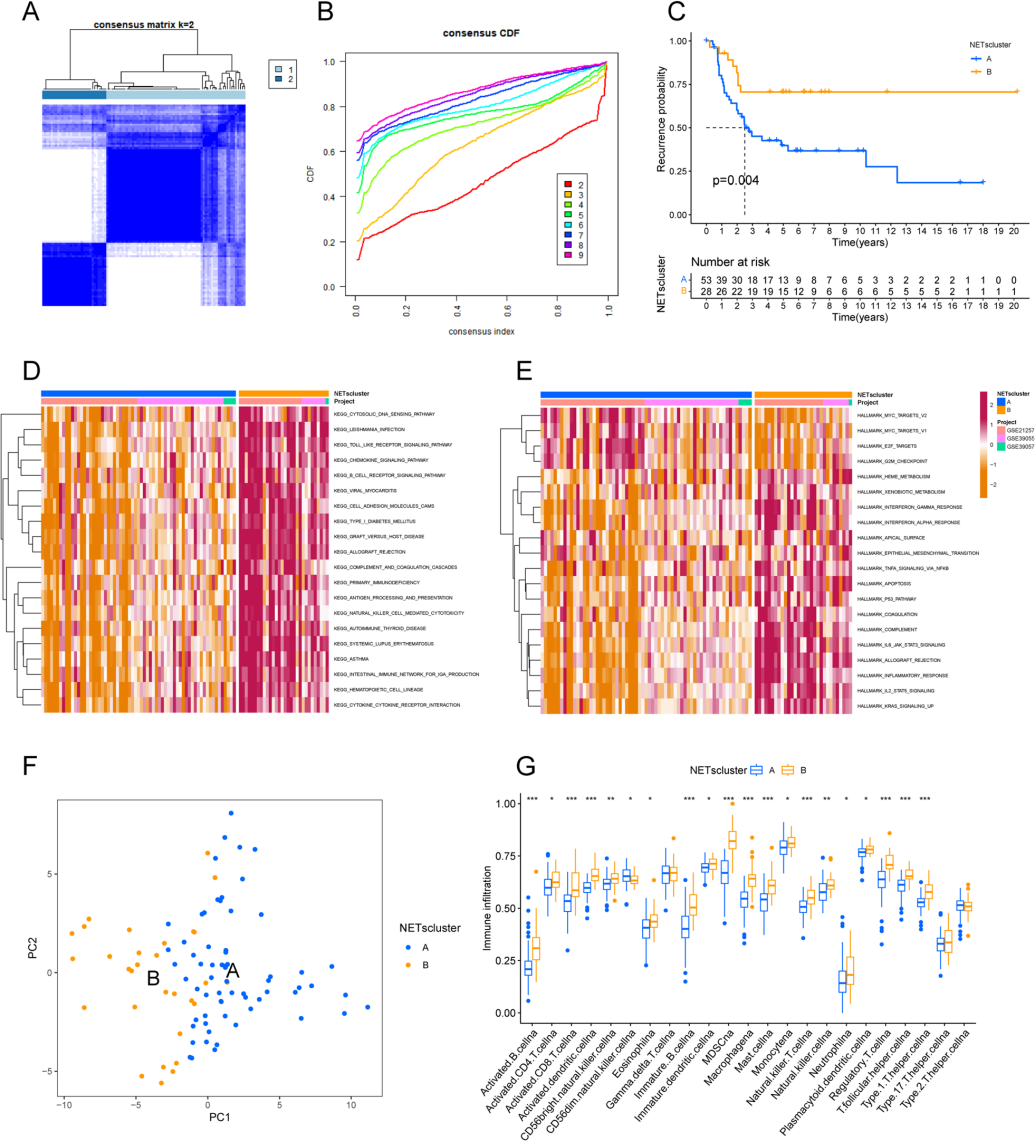
Identification of NETs cluster in OS. **A** and **B** show the consensus clustering matrix based on k = 2 of the metastasis-related NETs expressions, and **C** shows the Kaplan–Meier curves of the metastasis time in both NETs subtypes. **D** shows the GSVA scores of the KEGG pathway in both NETs subtypes. The red color indicates the activation pathway and the blue color indicates the inhibition pathway. **E** shows the GSVA scores of the Hallmark pathway in the two NETs subtypes. **F** shows the principal component analysis of the two NETs subtypes, showing the significant differences between the different subtypes. **G** shows the abundance of each infiltrating immune cell in the two NETs subtypes

Neutrophil extracellular traps (NETs) are reticular structures composed of DNA-histone complexes and proteins released by activated neutrophils. In addition to their key role in the neutrophil innate immune response, NETs are extensively involved in autoimmune diseases as well as other non-infectious pathological processes(Masucci et al. 2020). Based on the metascape platform (https://metascape.org/) to visualize the functional pathways of these NETs genes, the three most significantly enriched functional pathways are Neutrophil extracellular trap formation, Diabetic cardiomyopathy, and Platelet activation, and the interactions between different pathways were also obtained (Fig. 1A, B). Univariate Cox regression analysis and the STRING database (https://string-db.org/) were used to analyze the interaction network of these NETs genes (Fig. 1C, Table S1-2). The heatmap (Fig. 1D) demonstrates the NETs genes differentially expressed in the osteosarcoma non-metastatic and metastatic groups. We next performed consensus clustering analysis for all OS samples. The unsupervised clustering analysis revealed two different NETs clusters in OS (Fig. 2A, B). Interestingly, the time to metastasis was longer in OS patients with NETscluster-B than NETscluster-A (Fig. 2C).

### Construction of NETs-related gene clusters in OS

**Fig. 3:**
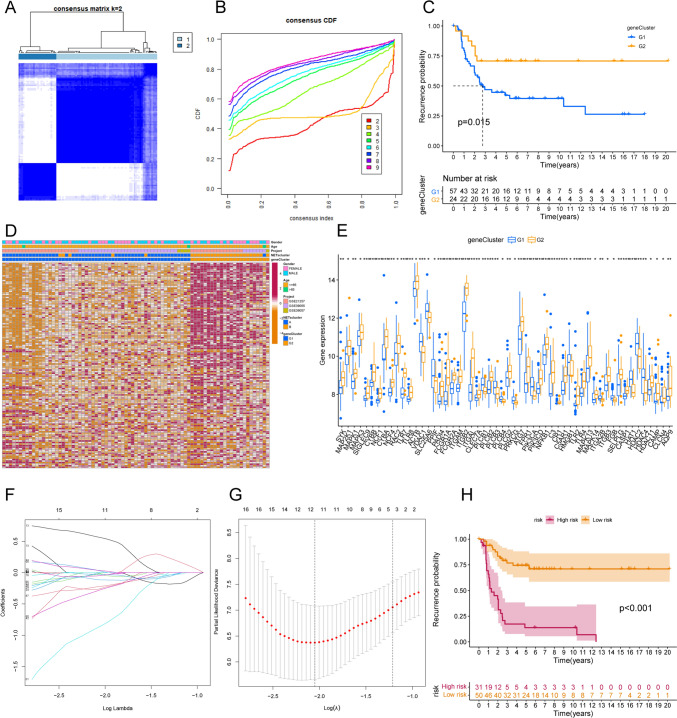
Identification of NETs-related gene clusters in OS. **A** and **B** show the consensus clustering matrix with k = 2 for the expression of the metastasis-associated NETs-associated DEG features, and **C** shows the Kaplan–Meier curves for the metastasis times of the two NETs-associated genotypes. **D** shows the heatmap of genotypes of metastasis-related NETs-associated DEG. NETs gene cluster, NETs cluster, dataset and age were used as patient annotations. **E** shows the expression of NETs genes in the two NETs gene clusters(*P < 0.05; **P < 0.01; *** P < 0.001). **F** shows the LASSO coefficient profiles for the NRGMS model, and **G** shows the confidence interval presentation under each lambda. **H** shows Kaplan–Meier transfer curves for all OS patients stratified according to the risk score

We used GSVA to further explore the biological behavior between different NETs subtypes, and the results showed that patients in the NETscluster-B group were significantly enriched in CYTOSOLIC_DNA_SENSING_PATHWAY, LEISHMANIA_INFECTION and TOLL_LIKE_RECEPTOR_ SIGNALING_PATHWAY pathways (Fig. 2D, Table S3), and significantly enriched in cancer signature pathways such as INFLAMMATORY_RESPONSE, IL2_STAT5_SIGNALING, and KRAS_SIGNALING_UP (Fig. 2E, Table S4). Principal component analysis indicated that OS patients were divided into two categories with significant differences after downscaling (Fig. 2F). The ssGSEA method was applied to further quantitatively assess the immune infiltration in different NETs subtypes. As shown in Fig. 2G, the vast majority of tumor-associated immune cells were more infiltrated in patients in the NETscluster-B group, such as activated CD8 T cells, natural killer cells, activated dendritic cells and neutrophils. We next applied the limma.R package to identify NETs-associated DEGs and univariate Cox regression analysis was performed to screen for prognostic metastasis NETs-associated DEGs (Table S5). Consensus clustering analysis revealed two NETs genotypes (named NETs-G1 and NETs-G2) based on the expression of prognostic metastasis NETs-associated DEGs (Fig. 3A, B). Survival analysis showed that NETs-G2 patients had a better prognosis for metastasis (Fig. 3C). The heatmap showed that NETs-G2 patients largely overlapped with the NETscluster-B subtype (Fig. 3D). And a higher expression of NETs was observed in the NETs-G2 subtype compared to the NETs-G1 subgroup (Fig. 3D, E).

### Construction of NETs-related gene metastasis signature

Lasso logistic regression model was built to assess the metastatic status of OS patients (Table S6). Five NETs-related genes were finally selected to generate the best risk score model (NRGMS) (Fig. 3F, G). We used the formula NETs risk score = Expression of TOMM40*0.594-Expression of CMTM2*2.683 + Expression of FH*1.233-Expression of TNFSF10*0.638-Expression of ETS2*1.049 to further calculate the risk score for each patient. Patients were then divided into high and low-risk groups using median risk. We found that the time to recurrence was much lower in the high-risk group than in the low-risk group (Fig. 3H), suggesting that a higher risk score is associated with a higher probability of metastatic recurrence in OS patients.

### Validation of the validity of NETs-related gene metastasis signature

**Fig. 4:**
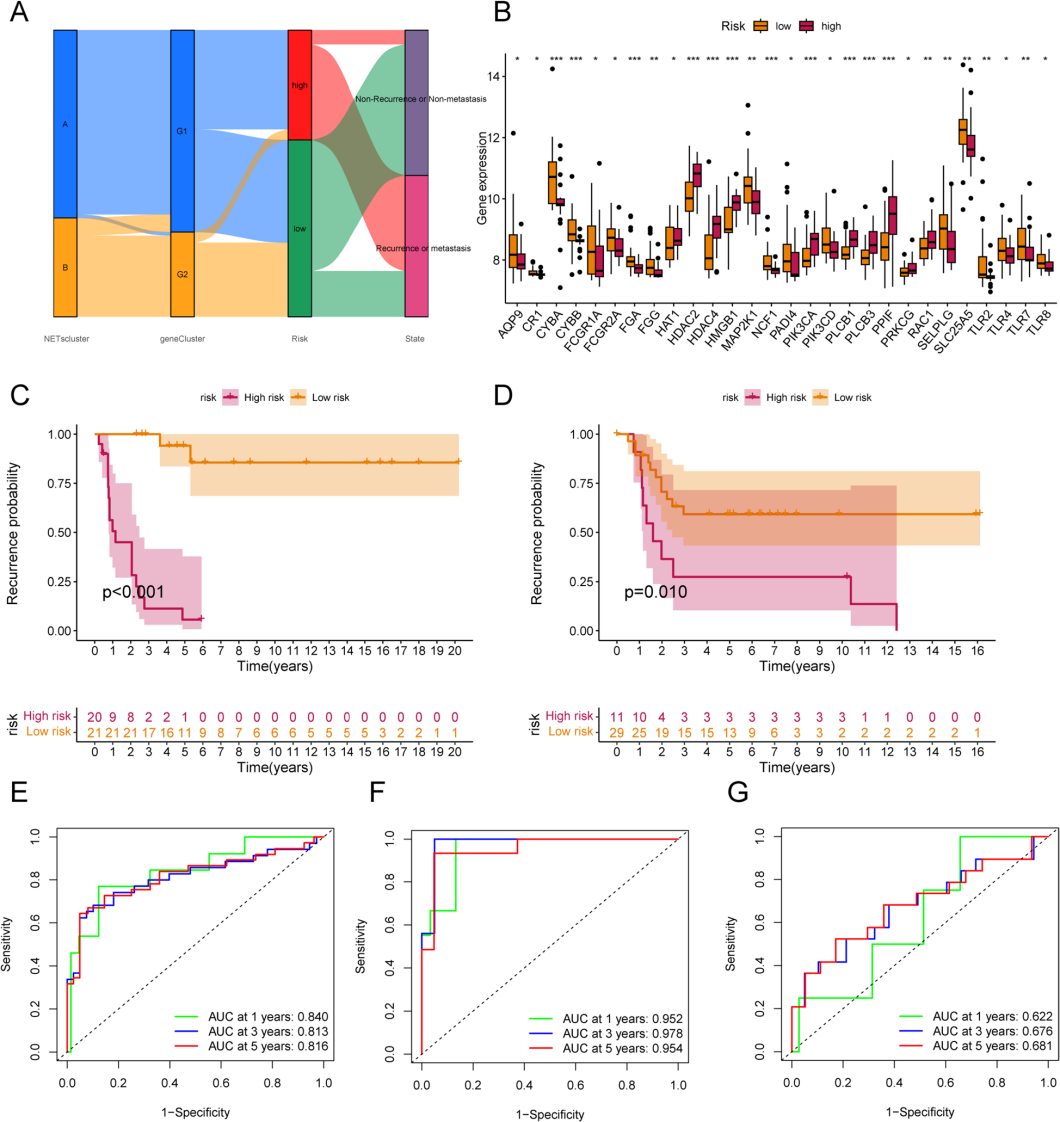
Construction of the transfer risk model associated with NETs. **A** shows the alluvial plot of NETs grouped in groups with different NETs subtypes, NRGMS and metastasis status. **B** shows the expression of NETs in the high and low-risk groups (*P < 0.05; ** P < 0.01; *** P < 0.001). **C**, **D** shows Kaplan–Meier metastasis curves for OS patients stratified according to risk score in the training and validation cohorts. **E**–**G** shows time-dependent ROC analysis of 1-year, 3-year, and 5-year OS prediction for OS patients in the total cohort, training cohort, and validation cohort

**Fig. 5:**
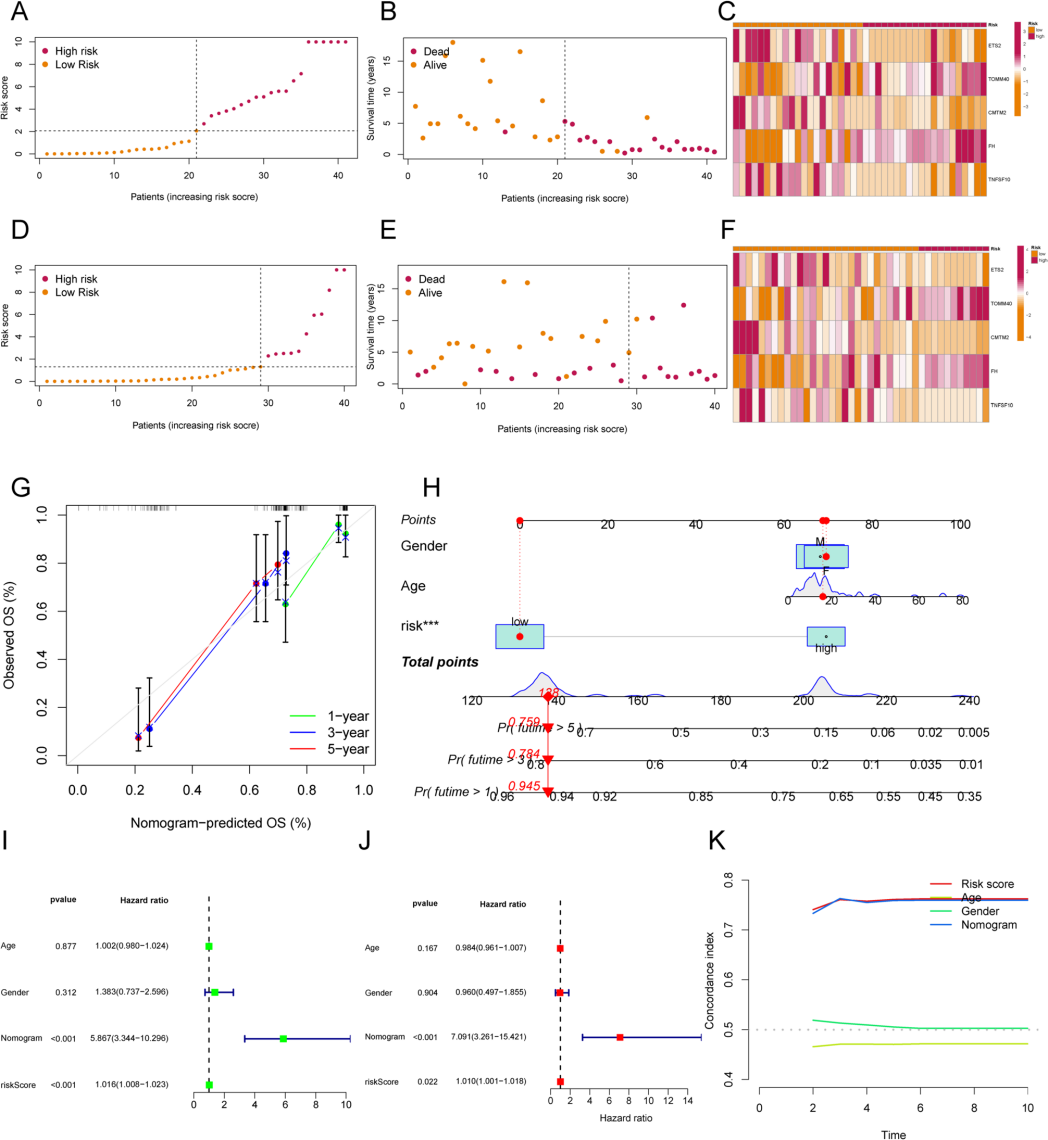
Test of the NETs-related transfer risk model. **A**, **D** shows the distribution of risk scores in the training and validation cohorts. **B**, **E** shows the distribution of time to metastasis for patients in the training and validation cohorts. **C**, **F** shows the heatmap depicting the expression of ETS2, TOMM40, CMTM2, FH and TNFSF10 between the high and low-risk groups in the training and validation cohorts. **G** shows the calibration curves for predicting the time to metastasis in OS patients at 1, 3 and 5 years based on the column line graphs. **H** shows column line plots predicting 1-year, 3-year and 5-year overall survival in OS patients based on NRGMS and other clinicopathological features. **I** shows the forest plot of NRGMS, Nomogram and other clinical characteristics of OS patients derived by univariate analysis. **J** shows the forest plots of NRGMS, Nomogram and other clinical characteristics of OS patients derived by multivariate analysis. **K** shows the consistency curves of NRGMS, Nomogram and other clinical characteristics of OS patients

Figure 4A shows the relationship between NETs cluster, NETs gene Cluster and NRGMS. We found that NETs cluster-B subtype in NETs-G2 subpopulation is associated with low risk, and low risk corresponds to a lower probability of metastatic recurrence. Figure 4B shows the differences in NETs genes between high and low-risk groups. Next, we divided the OS cohort into a training cohort (*N* = 41) and a validation cohort (*N* = 40). In both the training and validation cohorts, OS patients with high risk predicted a shorter time to metastasis than those with low risk (Fig. 4C, D). The time-dependent ROC curves also demonstrated the stability of the NRGMS model in the total cohort, the training cohort and the validation cohort (Fig. 4E-G). The distribution of the NRGMS model in the training and validation cohorts is shown in Fig. 5A, D. The scatter plot reveals that the metastasis rate of OS patients increases with increasing NRGMS levels (Fig. 5B, E). We also observed a significantly high expression of TOMM40 and FH in the high-risk group (Fig. 5C, F). We further developed a nomogram metastasis prediction model (Fig. 5H) to predict the metastatic status of OS patients. With the total score, we could predict the metastasis rate of OS patients at 1, 3 and 5 years. The OS curves of the column line graphs validate the robustness of the model (Fig. 5G). We combined age, sex, NRGMS and nomogram in Cox regression analysis, and we found that NRGMS and nomogram were poor independent prognostic factors in both univariate and multivariate independent prognostic analysis (Fig. 5I, J). Consistency score plots indicated that our nomogram and NRGMS had high stability (Fig. 5K).

### Immune characteristics and chemotherapy prediction in different risk subgroups

**Fig. 6:**
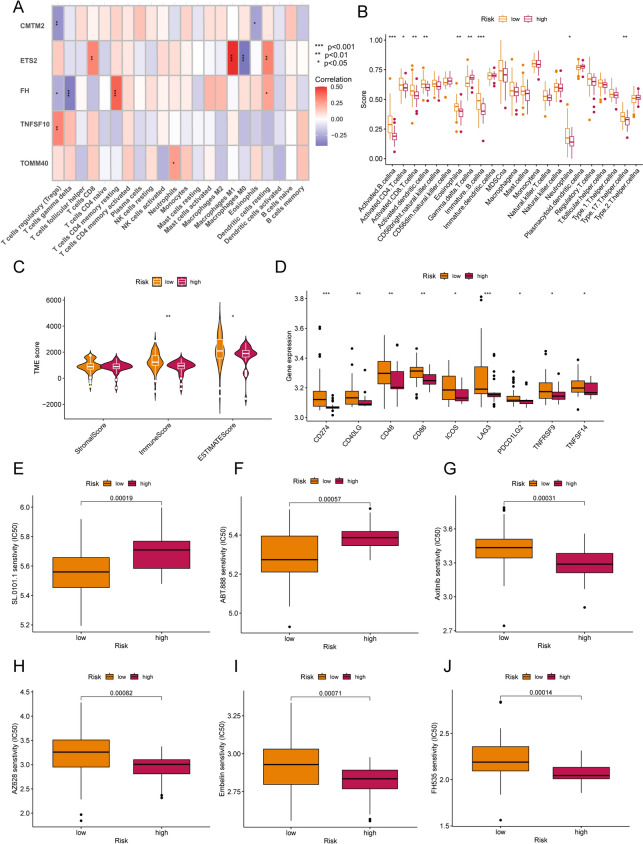
Immune infiltration and chemotherapy sensitivity analysis. **A** heatmap depicts the correlation between ETS2, TOMM40, CMTM2, FH, TNFSF10 and 22 immune infiltrating cells expression. **B** shows the comparison between high and low-risk groups in the expression of 22 immune infiltrating cells (*P < 0.05; ** P < 0.01; *** P < 0.001). **C** shows the levels of stromal, immune and ESTIMATE scores in different NRGMS groups according to the ESTIMATE algorithm. **D** shows the expression of immune checkpoints in the high and low-risk groups. The drugs associated with the high and low-risk groups of the model were visualized and **E**–**J** shows the box plot of IC50 expression in the high and low-risk groups for SL.0101.1, ABT.888, Axitinib, AZ628, Embelin and FH535, respectively

The CIBERSORT algorithm further investigated the level of immune cell infiltration in different NRGMS risk subgroups. We found a strong positive correlation of ETS2 expression with CD8 T cells, M1-type macrophages and resting dendritic cells, and a strong negative correlation with M0 macrophages (Fig. 6A, Table S7). In addition, ssGSEA analysis showed that the vast majority of tumor-associated immune infiltrating cells were more infiltrated in the low-risk group (Fig. 6B). Next, we compared the Stromal Score, Immune Score and ESTIMATE Score of the high and low-risk groups using the ESTIMATE algorithm (Table S8). we found that OS patients with low-risk had higher Immune Score and ESTIMATE Score (Fig. 6C). In addition, we found that the majority of immune checkpoints were upregulated in low-risk compared to the high-risk group (Fig. 6D), which may contribute more to the response to immunotherapy. Chemotherapy drug sensitivity analysis showed that OS patients with low-risk were more sensitive to drugs such as SL.0101.1 and ABT.888 (Fig. 6E, F), while OS patients with high-risk were more sensitive to Axitinib, AZ628, Embelin and FH535 (Fig. 6G–J), which could guide better clinical dosing in patients who have developed osteosarcoma metastases.

### Pan-cancer analysis of TOMM40 and FH

**Fig. 7:**
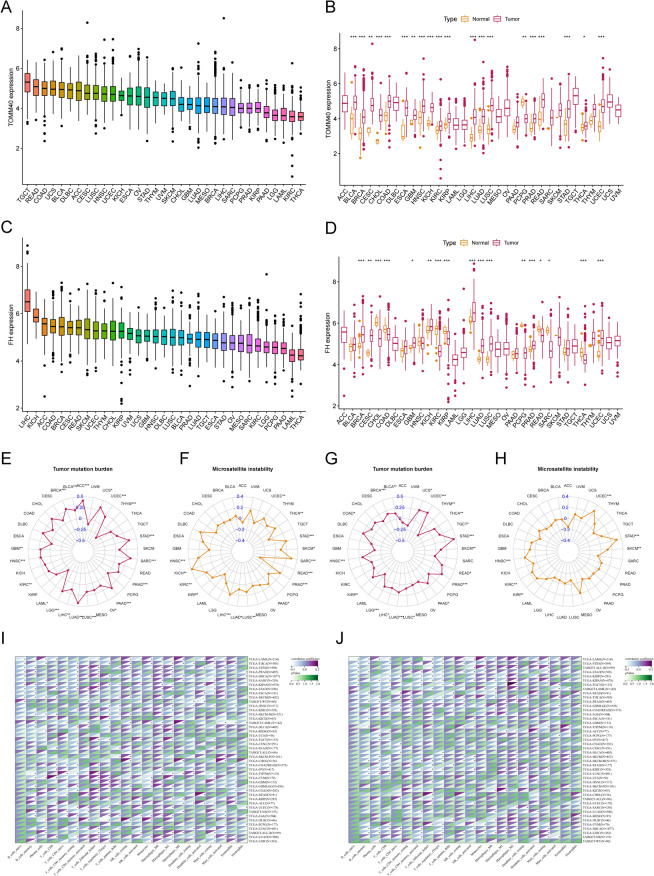
Pan-cancer analysis of TOMM40 and FH. **A**, **C** shows the expression of TOMM40 and FH in 33 cancers and **B**, **D** shows the expression of TOMM40 and FH in pan-cancer and normal tissues. **E**, **G** shows the correlation radar plots of tumor mutational burden of TOMM40 and FH in pan-cancer. **F**, **H** are the correlation radar plots of microsatellite instability of TOMM40 and FH in pan-cancer. **I**, **J** shows the co-expression analysis of TOMM40 and FH with immune cells in pan-cancer. (*P < 0.05, ** P < 0.01, *** P < 0.001)

To further analyze the important roles of TOMM40 and FH in other malignancies, we performed a pan-cancer analysis of TOMM40 and FH. Figure 7A, C shows the expression of TOMM40 and FH in 33 cancers, with the highest expression of TOMM40 in tenosynovial giant cell tumor (TGCT) and the highest expression of FH in liver cancer (LIHC). In addition, in most cancers, TOMM40 and FH expression differed significantly between tumor tissue and normal paracancerous tissue (Fig. 7B, D). TOMM40 and FH were associated with TMB and MSI in a range of cancers (Fig. 7E–H). We also performed the co-expression analysis of TOMM40 and FH with immune-related cells, and TOMM40 and FH could affect immune cell infiltration in pan-cancer (Fig. 7I, J).

### In vitro functional validation

**Fig. 8:**
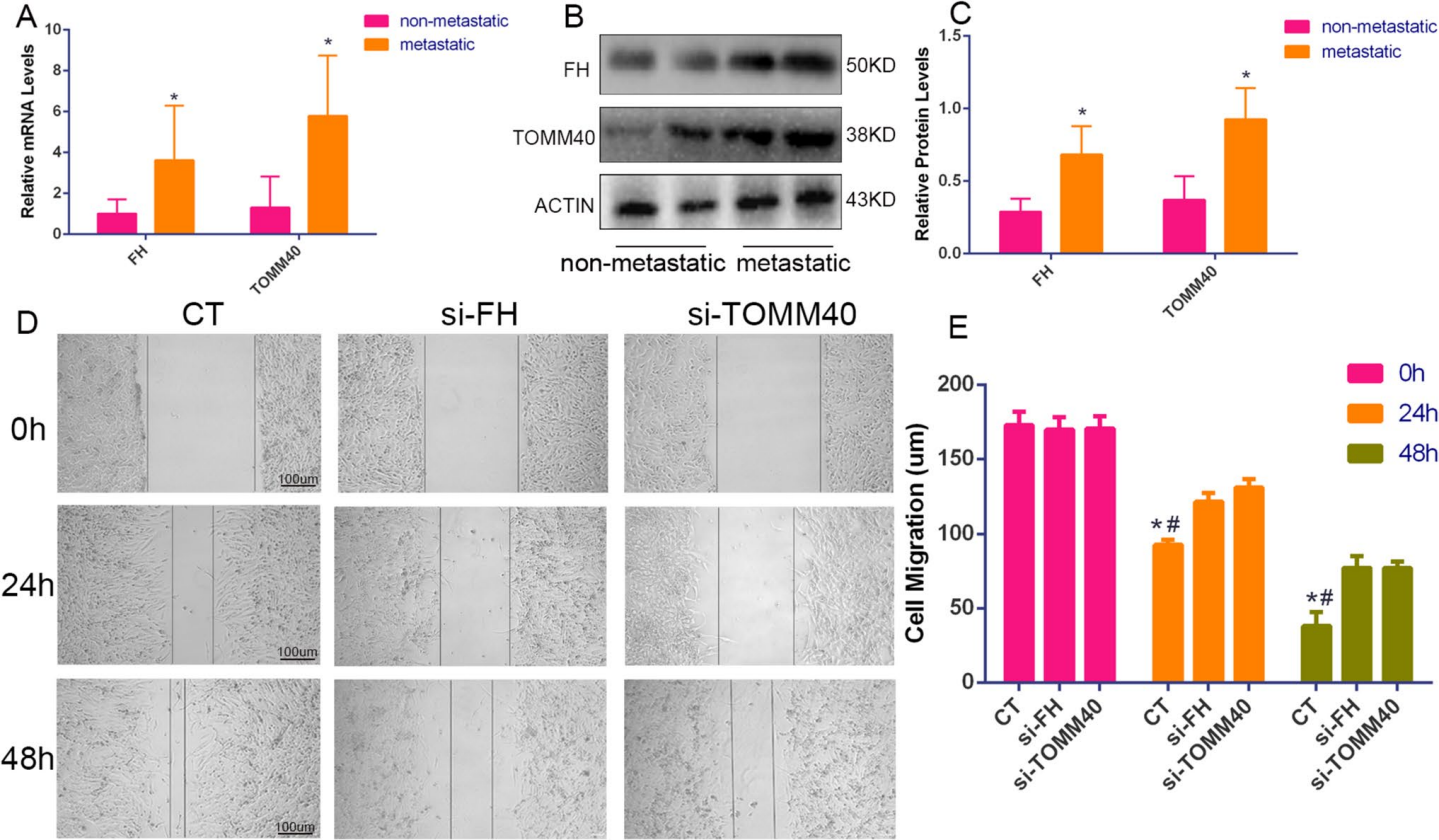
In vitro experimental validation. **A** shows the differences in RNA expression levels of FH and TOMM40 in the Osteosarcoma metastasis group and non-metastasis group tissues. **B**, **C** shows the difference in protein expression levels between FH and TOMM40 in the Osteosarcoma metastasis group and non-metastasis group tissues. **D**, **E** shows the scratch test of osteosarcoma cell lines after si-FH and si-TOMM40. Data on the distance between the left and right ends of the scratches were made at 0 h, 24 h and 48 h under the microscope, respectively

We found that TOMM40 and FH affect most malignancies. However, there are few studies on the mechanism of action of TOMM40 and FH in osteosarcoma. We further performed in vitro experiments to verify the expression of the two genes. qPCR results showed significant differences in the expression of TOMM40 and FH mRNA in the metastatic and non-metastatic groups of osteosarcomas (Fig. 8A). According to the Western blot results, the expression of TOMM40 and FH was higher in the metastatic tissues of osteosarcoma than in the non-metastatic group (Fig. 8B, C). In addition, we performed scratch assay results showing that TOMM40 and FH downregulation inhibited the migration ability of SW1353 cell lines (Fig. 8D, E).

## Discussion

Osteosarcoma is a common cancer in adolescents with high malignancy, high rate of recurrence and metastasis, and poor prognosis (Somarelli et al. 2020). The five-year survival rate of osteosarcoma is significantly reduced once distant metastases occur. Once osteosarcoma develops distant metastases, its five-year survival rate decreases dramatically, so metastatic recurrence of osteosarcoma is often associated with death (Zhang and Guan 2018). The prognostic indicators are currently focused on the number of patients with osteosarcoma. Current prognostic indicators are focused on assessing the prognosis of OS patients in clinical workups, and osteosarcoma metastatic features cannot be accurately represented in these models. Therefore, there is an urgent need to find reliable molecular biomarkers that can predict prognostic metastasis in OS to improve the survival of OS patients. Several studies have now demonstrated that NETs play a key role in the tumor immune microenvironment and contribute to tumor migration, invasion, and distant metastasis in different ways (Ireland and Oliver 2020; Demkow 2021). NETs protect tumor cells from interactions with neighboring antitumor immune cells such as NK cells and CD8 + T cells, further influencing the tumor immune landscape and tumor response to immunotherapy (Ireland and Oliver 2020; Teijeira et al. 2020). However, no studies have yet reported how NETs are involved in the migration, invasion and metastasis of osteosarcoma. Therefore, it is crucial to explore the relevance of a NETs-related OS metastasis prediction model based on the immune microenvironment of OS patients. Our study aims to characterize NETs gene expression patterns from a molecular subtype perspective and explore the immunotherapeutic value of NETs patterns in OS, hoping to provide a new direction for metastasis and treatment of OS patients and improve patient survival.

In the present study, we first identified 97 NETs genes in the OS gene profile and performed functional visualization through the online site mentioned above, where we found significant enrichment in Diabetic cardiomyopathy and Platelet activation pathways in addition to neutrophil-specific pathways. We identified 24 NETs genes differentially expressed in the OS metastatic and OS non-metastatic groups and divided OS patients into two groups using unsupervised clustering analysis, which showed significant differences in metastasis rates by subtype according to prognostic analysis, with a higher metastasis rate in the NETscluster-A group. In addition, immune infiltration analysis also showed that the majority of tumor immune-related cells (e.g., CD8T cells, NK cells, neutrophils, etc.) were also less infiltrated in the NETscluster-A group, which further suggested that the NETscluster-A group had a higher risk of metastasis. Next, we used GSVA to explore the differences between the KEGG pathway and the cancer HALLMARK pathway between the two NETsclusters, and we found that the vast majority of immune pathways were significantly enriched in the NETscluster-B group, while HALLMARK pathways such as (MYC_TARGETS_V2, MYC_TARGETS_ V1, E2F_TARGETS) were significantly down-regulated. It is well known that MYC oncoprotein and E2F transcription factors are key regulators of cell proliferation (Müller and Helin KJBeBA-RoC 2000; Cole and McMahon 1999), which suggests that the low invasiveness of the NETscluster-B group, and low metastasis are associated. We further compared transcriptomic differences between the two NETs groups and identified two subgroups of NETs-related genes. The two subgroups also showed significant differences in metastatic recurrence rate and NETs gene expression. Finally, we developed a quantitative system, NRGMS, to assess the NETs expression patterns of individuals. Our study found that higher NRGMS tended to be associated with osteosarcoma metastasis, suggesting that NRGMS are a negative indicator of OS. Further comparison of immune infiltration levels, immune fraction, and immune checkpoint expression between high and low-risk groups revealed that more immune cells were infiltrated and immune fraction and immune checkpoint expression were higher in the low-risk group, reflecting a better prognosis in the low-risk group.

In the present study, we applied TOMM40, CMTM2, FH, TNFSF10 and ETS2 to construct our NRGMS-related risk score. Several studies have reported that translocase of the mitochondrial outer membrane 40 (TOMM40) gene may increase the risk of developing Alzheimer's disease (AD) (Lee et al. 2021; Zeitlow et al. 2017). In addition, the TOMM40 gene has been strongly associated with human health and aging (Chen et al. 2022). Recently, a novel receptor on NK lymphocytes was reported to bind to TOMM40 on K562 leukemia cells, thereby initiating cytolysis, which may be related to the promotion of tumor cell growth (Alandejani et al. 2022). Numerous studies have shown that CMTM2 gene expression is associated with markers of the EMT process (Zhang et al. 2012). Xuefeng Guo found that CMTM2 was downregulated in HCC tissues by immunohistochemistry, which is consistent with our study (Guo et al. 2020). It has also been reported that inhibition of Akt activation and an increase in AR protein levels may be one of the mechanisms of enhanced CMTM2-mediated AR transactivation (Liu et al. 2009). FH heterozygous mutations predispose to FH gene tumor susceptibility syndrome, characterized by cutaneous smooth muscle tumors, uterine smooth muscle tumors (leiomyosarcomas), or renal tumors. However, no studies have reported an association between FH and the development of invasive osteosarcoma.

Numerous studies have reported an association between TNFSF10 and the progression of osteosarcoma (Kamihara et al. 2020). The findings of Yoo Jane Han suggest that TNFSF10 plays an important role in regulating the antiviral immune response in triple-negative breast cancer (TNBC) (Han et al. 2022). One study identified ETS2 inhibition as a potential therapeutic target for p53 mutant osteosarcoma (Pourebrahim et al. 2017). Interestingly, this is in contrast to our study of ETS2 inhibition in NRGMS, where an important function of ETS2 has been shown in numerous studies to be cell cycle protein D1 promoter regulation, thereby shortening the cell cycle and promoting tumor growth (Albanese et al. 1995; Fry and Inoue 2018). Given that TOMM40 and FH are upregulated in the osteosarcoma metastasis group, we further analyzed the pan-cancer characteristics of TOMM40 and FH. In most cancers, TOMM40 and FH were upregulated in the tumor group, suggesting that TOMM40 and FH play an important role in malignant proliferation and invasion. However, unfortunately, no relevant studies are showing the relationship between TOMM40 and FH and osteosarcoma. Therefore, we performed cell function validation of TOMM40 and FH. Through experimental validation analysis, TOMM40, FH RNA expression was shown to be significantly different in Osteosarcoma metastasis group and non-metastasis group tissues. Western blot further demonstrated a higher expression of TOMM40, FH in the metastatic group of osteosarcoma patients. Meanwhile, wound healing showed that si-TOMM40 and si-FH reduced the invasive ability of osteosarcoma cell lines. Simple in vitro cell models are very different from the real tumor ecosystem. To decipher the full picture and predict the dynamic evolution of tumor diseases, variables such as the cellular and non-cellular composition of the tumor microenvironment (TME) and its spatiotemporal distribution must be considered. Tippett et al. recognized that survival in osteosarcoma (OS) has been stagnant because patients are often resistant to neoadjuvant MAP chemotherapy, including high-dose methotrexate, doxorubicin (doxorubicin), and platinum (cisplatin). Therefore, it is essential to establish cell models related to MAP resistance as a tool for drug discovery to solve this problem (Tippett et al. 2023). Furthermore, some studies attempt to combine in vitro 3D models with in vivo models to allow more robust and reliable studies to develop more effective anti-cancer therapies, which will bring preclinical research to the next stage (Miserocchi et al. 2023). The application of these strategies can better verify the feasibility of our results in the future.

Of course, there are still some limitations to our study. First, the number of samples was small and external cohorts were missing for validation, and second, in addition to TOMM40 and FH, other genes associated with features should also be validated at the cytological level. At the same time, the specific mechanism of hub genes in the regulation of osteosarcoma metastasis is still lacking, and further in vivo and in vitro experiments are needed to verify these results. In this paper, we constructed the first osteosarcoma metastasis risk signature consisting of 5 NETs-associated genes. In addition, we found that this signature was closely associated with immune cell infiltration and immune checkpoint expression in osteosarcoma patients. Our study provides a new approach to predicting the metastatic profile of osteosarcoma patients and identifies new therapeutic targets.

### Supplementary Information

Below is the link to the electronic supplementary material.

Supplementary file1 (ZIP 501 KB)

## Data Availability

The datasets analyzed during the current study are available in the Gene Expression Omnibus (https://www.ncbi.nlm.nih.gov/geo/query/acc.cgi?acc=gse21257, https://www.ncbi.nlm.nih.gov/geo/query/acc.cgi?acc=GSE39055, https://www.ncbi.nlm.nih.gov/geo/query/acc.cgi?acc=GSE39057) and the UCSC Xena database (https://xenabrowser.net/datapages/?cohort=TCGA%20Pan-Cancer%20(PANCAN)&removeHub=https%3A%2F%2Fxena.treehouse.gi.ucsc.edu%3A443). All data used in this study are available from public databases and can be downloaded by all through the link above.
